# Causes of failed anterior cruciate ligament reconstruction: A retrospective case series

**DOI:** 10.1097/MD.0000000000041480

**Published:** 2025-02-07

**Authors:** Xiao Jin, Zihan Lin, Meiping Yang, Jinlong Zhao, Lingfeng Zeng, Jun Liu, Guihong Liang, Weiyi Yang, Jianke Pan

**Affiliations:** a Department of Chinese Medicine, The First Affiliated Hospital of Jinan University, Guangzhou, China; b The Second Clinical Medical College of Guangzhou University of Chinese Medicine, Guangzhou, China; c The Second Affiliated Hospital of Guangzhou University of Chinese Medicine (Guangdong Provincial Hospital of Chinese Medicine), Guangzhou, China; d The Research Team on Bone and Joint Degeneration and Injury of Guangdong Provincial Academy of Chinese Medical Sciences, Guangzhou, China.

**Keywords:** ACLR, anterior cruciate ligament reconstruction, case series, causes

## Abstract

**Rationale:**

The number of anterior cruciate ligament reconstruction (ACLR) surgeries in China is steadily increasing. To enhance the success rate of ACLR, it is crucial to understand the reasons for ACLR failure. The purpose of this study is to determine the primary reasons for ACLR failure and evaluate the technical skills associated with the revision procedure.

**Patient concerns:**

A retrospective clinical data analysis was performed for all patients who underwent anterior cruciate ligament revision surgery between January 2014 and September 2022. Each patient’s data set consisted of the 3 items listed below: standardized imaging data, medical records, and all arthroscopic images and recordings from the revision surgery.

**Diagnoses:**

A total of 65 patients underwent a failed ACLR surgery and then had to undergo revision surgery. Among these patients, the causes of revision were inappropriate tunnel placement (12, 18.75%), graft fixation problems (4, 6.25%), traumatic reinjury (35, 54.69%), graft failure (8, 12.5%), multiple ligament injuries (1, 1.56%), and infection (5, 7.81%).

**Interventions and Outcomes:**

In patients with inappropriate tunnel placement, the femoral canal deviated anteriorly in 6 cases, posteriorly in 4 cases, and the tibial canal deviated anteriorly in 2 cases. In patients with graft fixation failure, the loop plate was loose in 1 case, the screw was not screwed in 2 cases, and the metal guidewire was not pulled out in 1 case. Of patients who suffered traumatic reinjury, 24 suffered high-power trauma, whereas 11 suffered low-power trauma. Graft relaxation occurred in 3 cases, and absorption was noted in 5 cases among graft failure patients. Joint instability resulted from multiple knee ligament injuries, along with medial and lateral collateral ligament damage. In cases of infection, knee joint infection occurred in 3 cases, while 1 case involved wound infection combined with bone tunnel lysis, and another case involved a knee joint infection emerging after the revision procedure.

**Lessons:**

ACLR failure is associated with traumatic reinjury, inappropriate tunnel placement, graft failure, graft fixation problems, infection, and multiple ligament injuries. Particular emphasis should be placed on the precise positioning of bone tunnels during surgical procedures. Proper manipulation of the aforementioned influencing factors is crucial to the success rate and therapeutic efficacy of arthroscopic ACLR.

## 1. Introduction

Injuries to the anterior cruciate ligament (ACL) are frequent in sports, with a reconstruction rate of 74.6 per 100,000 in the United States.^[1]^ The anterior cruciate ligament reconstruction (ACLR) revision rate ranges from 0.7% to 10% due to trauma, poor postoperative recovery, or function limitations, which increase in tandem with the volume and advancement of ACLR surgery.^[2]^ The key to a successful revision surgery is determining why the primary reconstruction failed. According to a multicenter ACL revision study, compound factors were the primary cause of failure in the majority of cases (37%), followed by secondary trauma (32%), technical errors (24%), and biological factors (7%).^[3]^ The most frequent technical problems include inappropriate tunnel placement and orientation, improper graft sizes, hardware failures, unsolved multiple ligament injuries, and unidentified limb malalignment. Notably, while autografts generally exhibit effectiveness and minimal morbidity at the transplant site, allografts present a contrasting scenario. Research indicates that allografts can notably heighten the likelihood of a revision procedure, particularly among youthful and active patients, due to failed tendon-bone healing.^[1]^

To enhance the success rate of revision surgeries and minimize the chances of surgical errors, it is crucial to comprehend the causes of reconstruction failure. The main objective of this study is to analyze the primary reasons behind the failure of ACLR and evaluate the technical expertise required for the revision procedure.

## 2. Methods

### 2.1. Case designs and summaries

This study was designed as a retrospective case series study. The ACLR revision surgeries were performed from 2014 to April 2022 by experienced surgeons. Patients who underwent revision surgery after ACLR with complete case data were included, while primary ACLR cases were excluded. A total of 65 patients, including 52 men (80%) and 13 women (20%), underwent revision surgery due to ACLR failure between January 2014 and September 2022 (mean age, 30 years). The periods between the primary ACLR procedures and the revision surgeries varied from 2 days to 10 years. The former data was gathered based on the patients’ inpatient and follow-up records to evaluate the treatment received during revision surgery. The Ethical Committee of Guangdong Province Hospital of Chinese Medicine approved this study (ZE2022-291-01). And written informed consent was obtained from all participants.

### 2.2. Data collection and analysis

Retrospective clinical data analysis was performed for all patients who underwent ACL revision surgery. The data set of each patient comprised the 3 items listed below: standardized imaging data, medical records, and all arthroscopic images and recordings from the revision surgery. Each record contained the following information: age, sex, presence of an initial meniscus or other ligamental injury, when the primary ACLR was performed, graft type, fixation type, bone tunnel placement and direction, and whether combined injured meniscus and ligaments were repaired.

Three skilled sports medicine physicians evaluated the primary ACLR situation based on standardized imaging data and intraoperative exploration. Data regarding the revision procedure were collected, including graft type, fixation type, bone tunnel placement, size, and whether combined injured meniscus and ligaments were repaired. Complaints cited 3 types of factors as causes of admission: trauma (secondary trauma), unclear causes (poor recovery after original reconstruction), and postoperative infection. The reasons for ACLR failure were categorized as follows based on medical records, imaging data, and intraoperative explorations during the revision procedures: trauma factors, technical errors, biological factors, infections, fixation factors, and combined factors.

## 3. Results

### 3.1. Causes of revision after ACLR in 65 patients

Among 65 patients, the reasons for revision included inappropriate tunnel placement (n = 12, 18.75%), graft fixation problems (n = 4, 6.25%), traumatic reinjury (n = 35, 54.69%), graft failures (n = 8, 12.5%), multiple ligament injuries (n = 1, 1.56%), and infection (n = 5, 7.81%). Further details are provided in Table 1.

**Table 1:** Cause of primary ACLR failure.

Cause	Number	Details
Inappropriate tunnel placement	12	The femoral canal deviated anteriorly in 6 cases, posteriorly in 4 cases; and the tibal canal deviated anteriorly in 2 cases.
Graft fixation failure	4	The loop plate was loose in 1 cases; the screw was not screwed in 2 cases; and the metal guide wire was not pulled out in 1 case.
High-power traumatic reinjury	24	The injuries including playing basketball, football and badminton, jumping from high places, cycling, and collision of affected limbs.
Low-power traumatic reinjury	11	The injuries including sprains, slips and falls.
Graft failure	8	The graft relaxation in 3 cases and absorption in 5 cases.
Multiple ligaments injury	1	Multiple knee ligament injuries, combined with medial collateral ligament and lateral collateral ligament injuries.
Infection	5	Knee joint infection in 3 case, wound infection combining bone tunnel lysis in 1 case, knee joint infection after revision in 1 case.

ACLR = anterior cruciate ligament reconstruction.

### 3.2. Analysis of failed ACLR causes

#### 3.2.1. Severe secondary trauma

**Figure 1.**
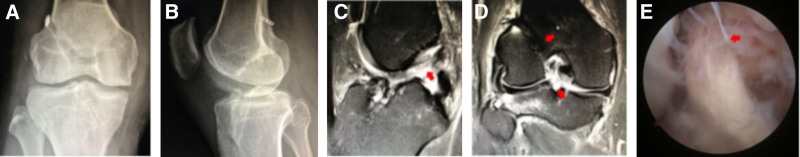
(A and B) DR: the tibial and femoral channels of the right knee were in good positions, and no significant expansion of the bone tunnel was observed. (C and D) MRI findings: the ACL graft ruptured near the femoral ending point, and the tendon in the femoral bone tunnel showed hypointensity. (E) Intraoperative picture: the ACL graft ruptured near the femoral ending point. ACL = anterior cruciate ligament.

Case 1 involved a 39-year-old male who underwent ACLR in 2014 (Fig. 1). Their postoperative recovery was good, enabling a return to sports. Eight years later, the graft ruptured again while playing basketball. During the revision procedure, the original graft ruptured from the femoral side, while the broken end morphology was intact. Subsequently, ACL revision was performed (autograft + allograft, 8 mm).

#### 3.2.2. Slight secondary trauma

**Figure 2.**
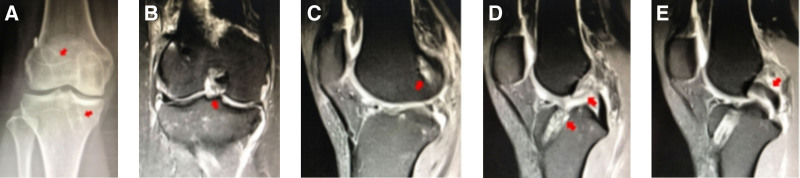
(A) DR: the tibial and femoral channels of the right knee were in good positions, albeit with slight enlargement of the bone tunnel. (B and C) MRI findings: the ACL was ruptured, and the signal of the tendon in the femoral tunnel was somewhat mixed when it was close to the joint cavity. (D and E) MRI findings: evidence of ACL rupture was observed, with the PCL demonstrating good morphology and tension; however, mixed signals were observed in the tendons within the tibial canal. ACL = anterior cruciate ligament, PCL = posterior cruciate ligament.

Case 2 involved a 31-year-old female who underwent ACLR in 2019 (autograft, 7 mm), along with a partial meniscectomy (Fig. 2). Following surgery, their postoperative recovery was satisfactory, enabling self-care. However, after 2 years, the graft experienced partial rupture due to a slip-and-fall incident. Consequently, ACL revision was performed (allograft, 8 mm) in combination with meniscoplasty.

#### 3.2.3. Inappropriate tunnel placement: anterior femoral tunnel

**Figure 3.**
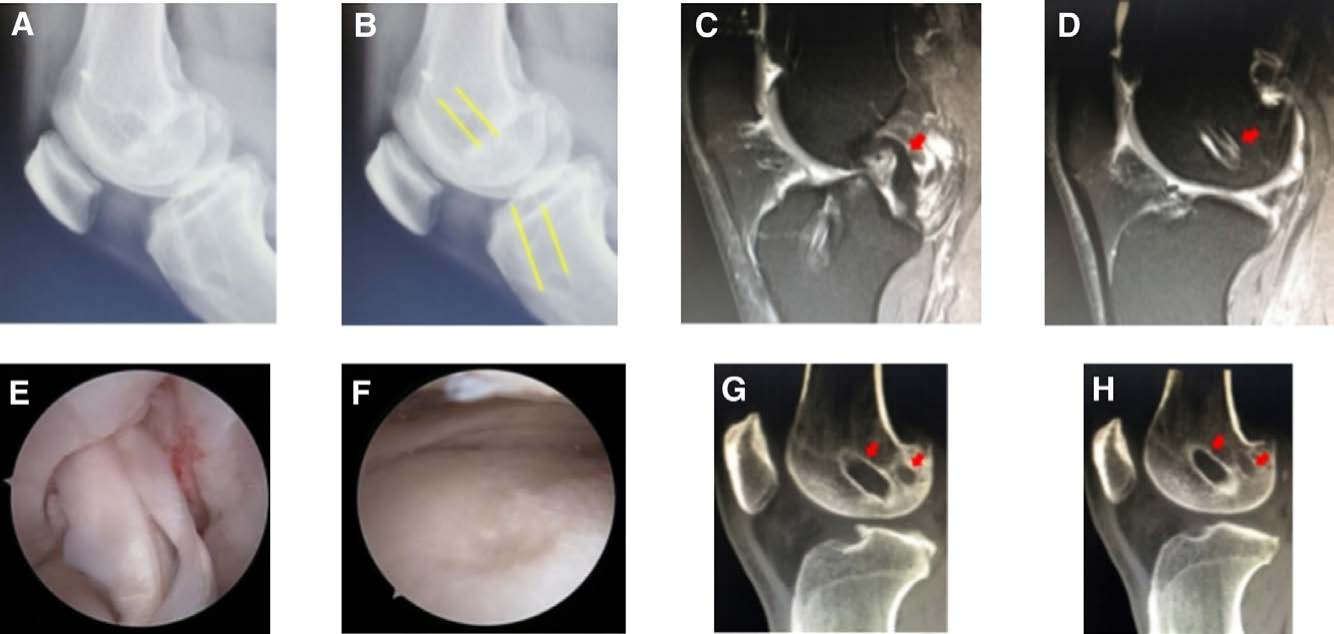
(A) DR: the tibial tunnel of the right knee was significantly enlarged, and the femoral tunnel was significantly anteriorly positioned. (B) The yellow line marks the location of the femoral and tibial tunnel, with the femoral tunnel situated anteriorly. (C and D) MRI findings: PCL relaxation exhibited a question mark configuration, ACL graft relaxation exhibited signal disorder, and the femoral tunnel was positioned significantly anterior. (E and F) Intraoperative findings: hyperosteogeny in the intercondylar fossa, ACL graft relaxation, and total knee degeneration. (G and H) CT image: reconstruction of the anatomic bone canal was performed post-operation. ACL = anterior cruciate ligament, PCL = posterior cruciate ligament.

Case 3 involved a 32-year-old male who underwent ACLR in 2012 (allograft, 8 mm), combined with partial meniscus suture (Fig. 3). Their postoperative recovery was poor, with continuous knee instability. In 2020, an MRI revealed posterior cruciate ligament (PCL) relaxation represented by a question mark-shaped sign, along with signal disorder observed in the ACL graft. Additionally, the MRI showed clear anteriorization of the femoral tunnel. ACL revision surgery was performed, and obvious joint degeneration was observed during the operation. The femoral bone canal was reconstructed behind the original bone canal during the operation, as corroborated by CT scans. The postoperative radiographs revealed anterior positioning of the femoral tunnel, prompting ACL revision surgery to relocate the tunnel backward (autograft + allograft, 7 mm).

#### 3.2.4. Inappropriate tunnel placement: anterior tibial tunnel

**Figure 4.**
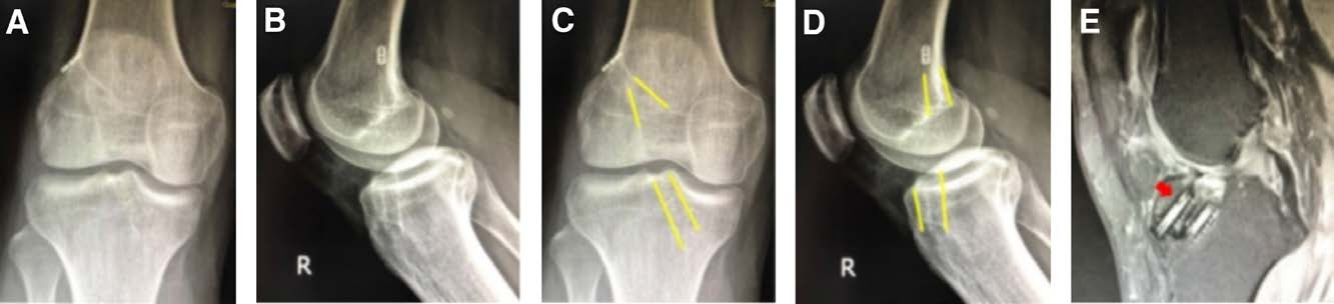
(A and B) DR: the tibial tunnel was in a good position; however, it appeared significantly anterior in the lateral view. (C and D) The yellow line marks the location of the femoral and tibial tunnels, with the tibial tunnel identified as being positioned anteriorly. (E) MRI image: at 4 months postoperatively, adjustment was made to the tibial tunnel position.

Case 4 involved a 28-year-old male who underwent ACLR in 2016 (Fig. 4). One year post-reconstruction, instability was noted in the knee joint, particularly on stairs. MRI findings indicated graft laxity, with the tibial tunnel identified as being positioned anteriorly. During the revision surgery, the residual ACL was restored, along with repositioning of the tibial tunnel posteriorly. Subsequently, another graft was transplanted (allograft, 6 mm).

#### 3.2.5. Inappropriate tunnel placement: anterior femoral tunnel and broken Milagro wire

**Figure 5.**
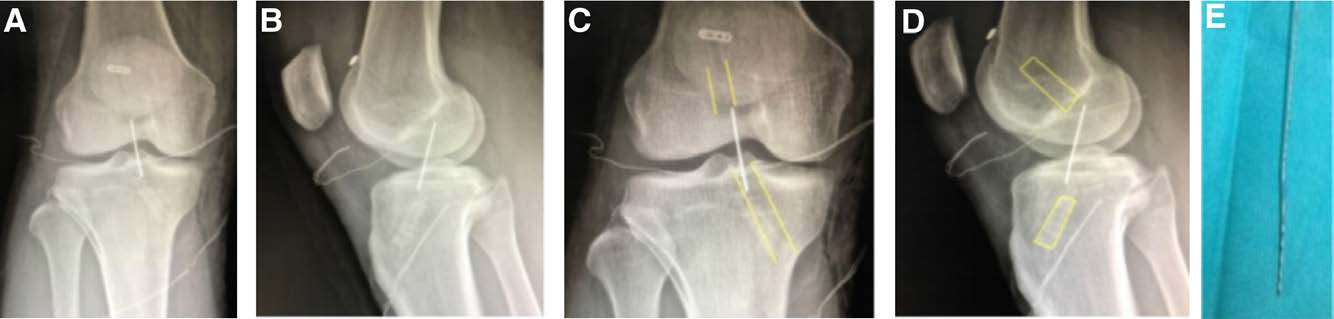
(A and B) DR findings: the titanium plate on the femoral side was in the suprapatellar capsule, the femoral bone canal was positioned anteriorly, and a broken Milagro guidewire was identified in the joint cavity. (C and D) The yellow line marks the location of the femoral and tibial tunnel, with the femoral tunnel situated anteriorly. (E) Broken guidewire that remained post-surgery.

Case 5 involved a 43-year-old male who underwent ACLR in 2022 (autograft, 8 mm), combined with partial meniscus suture (Fig. 5). Postoperative radiographs showed that the femoral tunnel was positioned anteriorly, and a broken Milagro wire was found within the joint space.

#### 3.2.6. Inappropriate tunnel placement: posterior femoral tunnel and displaced fixation plate

**Figure 6.**
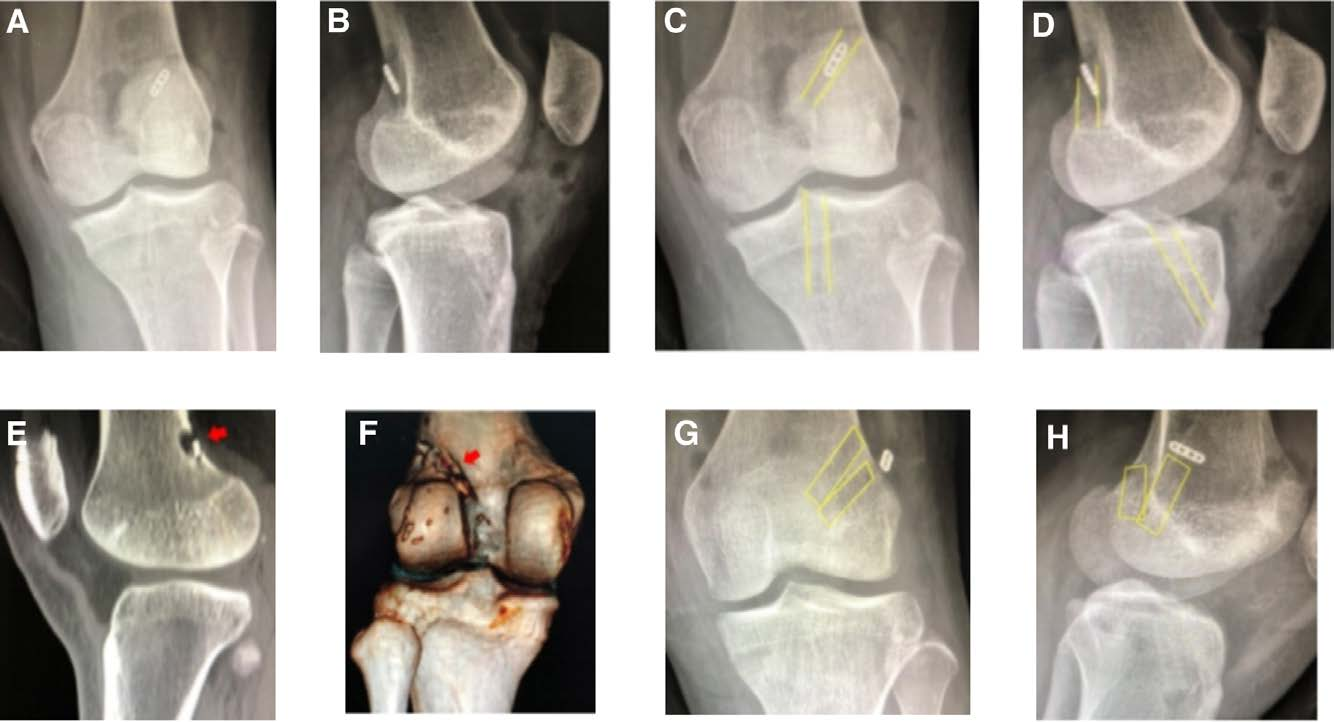
(A and B) DR findings: the femoral suspension EndoButton plate was displaced in the femoral tunnel, and the femoral tunnel was positioned posteriorly. (C and D) The yellow line marks the location of the femoral and tibial tunnel, with the femoral tunnel situated posteriorly. (E and F) CT findings: the femoral suspension EndoButton was displaced in the femoral tunnel, and the posterior wall of the femoral tunnel was broken. (G and H) DR results: the yellow line marks the location of the femoral and tibial tunnel, with the bone canal adjusted anteriorly to maintain the integrity of the posterior wall of the bone canal and increase the length of the bone tunnel.

Case 6 involved a 14-year-old female who underwent ACLR in 2019 (autograft, 7 mm) without meniscus management (Fig. 6). Postoperative radiographs revealed the posterior positioning of the femoral tunnel, with the fixation plate displaced into the tunnel. Subsequently, ACL revision surgery was performed to reposition the femoral tunnel anteriorly (autograft, 7 mm).

#### 3.2.7. Fixation failures

**Figure 7.**
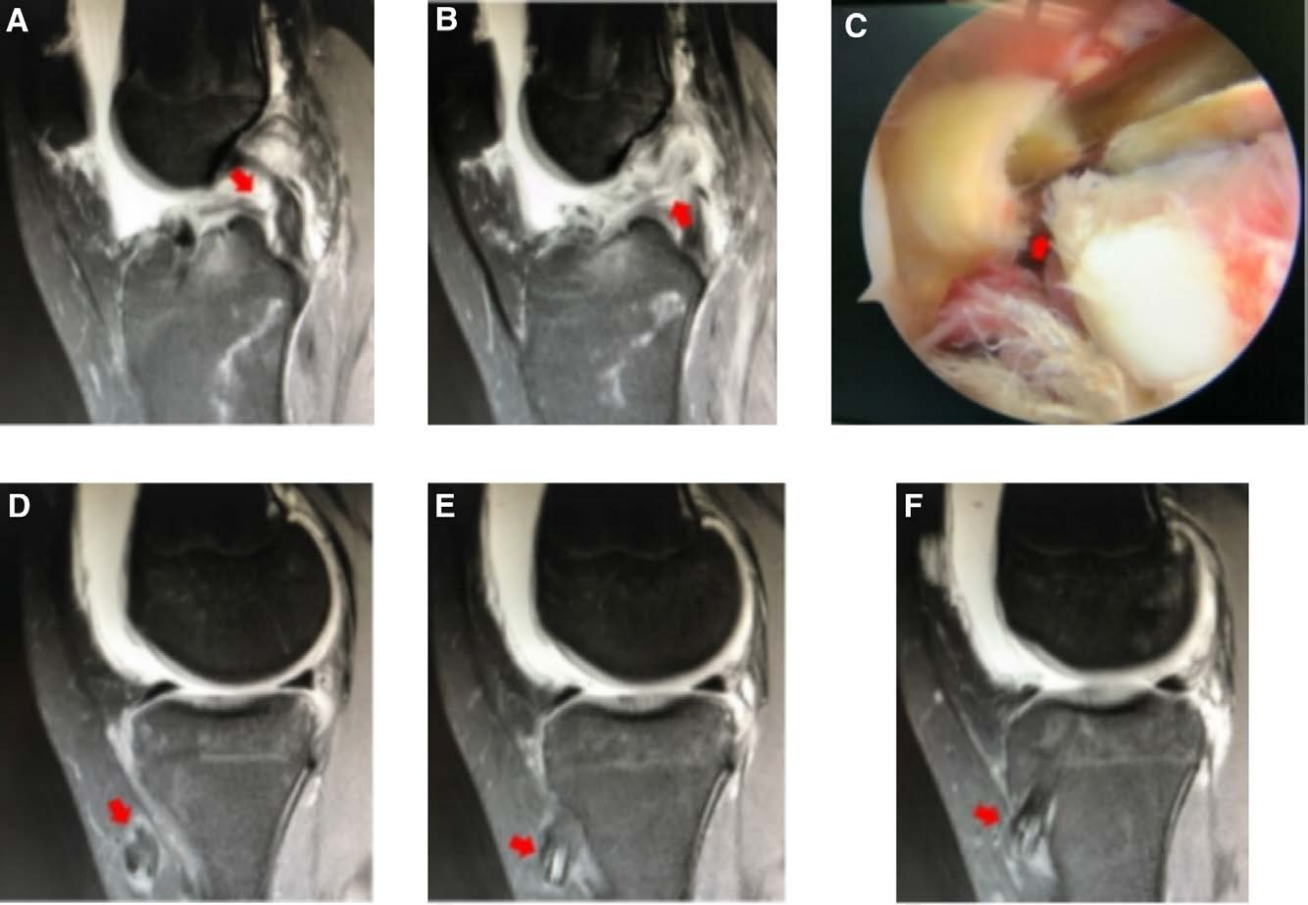
(A and B) MR: complete rupture of the ACL in the right knee. (C) Intraoperative image showing that the ACL graft had ruptured. (D–F) MRI findings showed that the tibial extrusion nail was not fully screwed into the bone canal. ACL = anterior cruciate ligament.

Case 7 involved a 15-year-old male who underwent ACLR in 2018 (allograft, 7 mm), combined with partial meniscectomy (Fig. 7). During follow-up, the patient reported feeling unstable, and radiographs revealed that the screw had dislodged from the tunnel. Subsequently, ACL revision surgery was performed (autograft, 8 mm).

**Figure 8.**
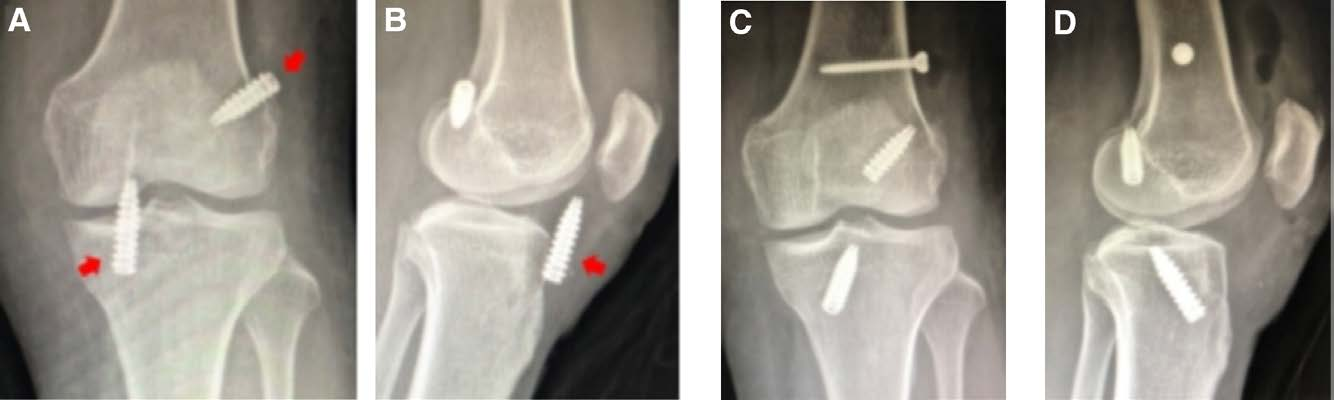
(A and B) DR: the femoral and tibial screws were not completely screwed into the tunnel. (C and D) DR: the screws fixed on the femoral and tibial sides were adjusted. Because the screw on the femoral side was not firmly fixed, an additional screw was used for compilation and fixation.

Case 8 involved a 55-year-old female who underwent ACLR in 2019 (allograft, 6 mm), combined with partial meniscus suture (Fig. 8). The postoperative radiographs showed that the screws were out of position. Subsequently, during the revision surgery, the 2 screws were repositioned and secured.

#### 3.2.8. Broken Kirschner wire in the tibial tunnel

**Figure 9.**
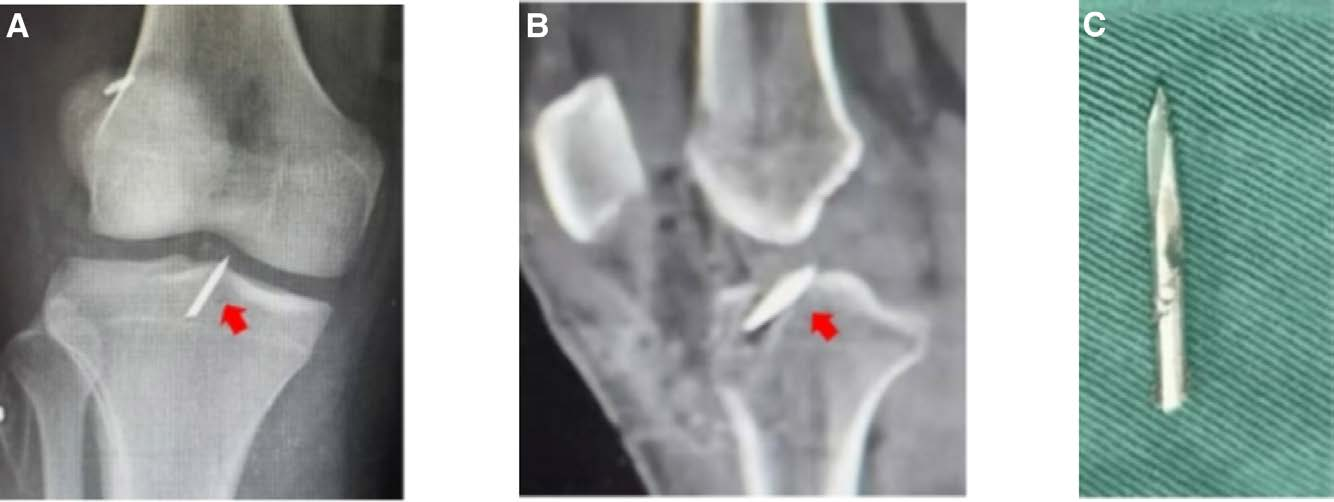
(A and B) Broken steel wire left within the joint space. (C) The wire was removed during the revision procedure.

Case 9 involved an 18-year-old male (Fig. 9). Their postoperative radiographs showed a broken Kirschner wire in the tibial tunnel. During the revision procedure, the broken wire was removed.

#### 3.2.9. Ligamentization failure and graft absorption

**Figure 10.**
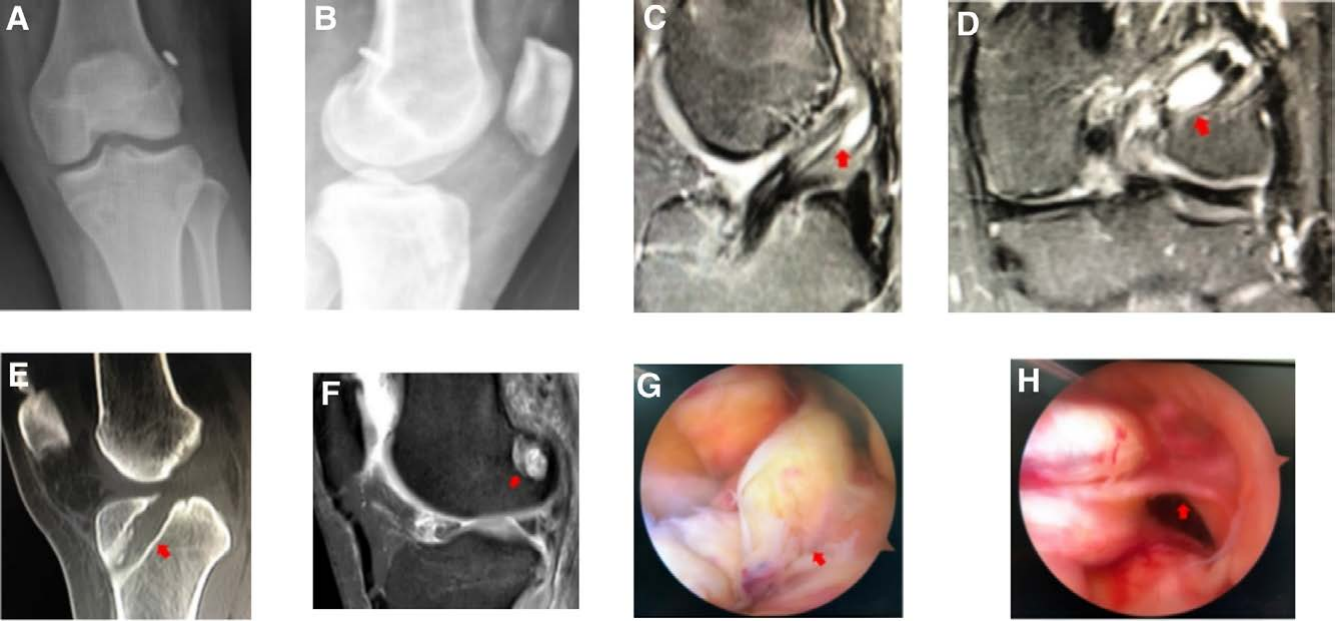
(A and B) DR findings: The EndoButton plate was not tightly positioned close to the periosteum, and the femoral and tibial tunnels were in good positions. (C and D) MRI findings 6 months after the operation: the femoral tunnel portal and tendon showed relatively high signal intensity. In particular, the proportion of high signal intensity in the femoral tunnel exceeded 50%, indicating ligamentization failure in the tendon. (E) CT showed that the tibial and femoral tunnels were significantly enlarged, with locally hardened edges. (F) MRI after the second injury revealed a tear in the posterior horn of the medial meniscus. (G and H) Intraoperative exploration revealed the presence of a fatty ACL and tendon vacancy in the femoral tunnel. Additionally, the shape and tension of the lateral meniscus were deemed satisfactory. The RAM region of the medial meniscus exhibited a tear resembling a barrel handle, which was subsequently repaired and secured with sutures. ACL = anterior cruciate ligament.

Case 10 involved a 22-year-old female who underwent ACLR in 2020 (allograft, 8 mm) without meniscus management (Fig. 10). Two years after reconstruction, the graft tore again during a basketball game. The MRI scan showed a high signal within the bone tunnel. Subsequently, intraoperative exploration revealed allograft absorption, prompting ACL revision surgery (autograft, 8 mm).

#### 3.2.10. Rejection reaction and graft absorption

**Figure 11.**
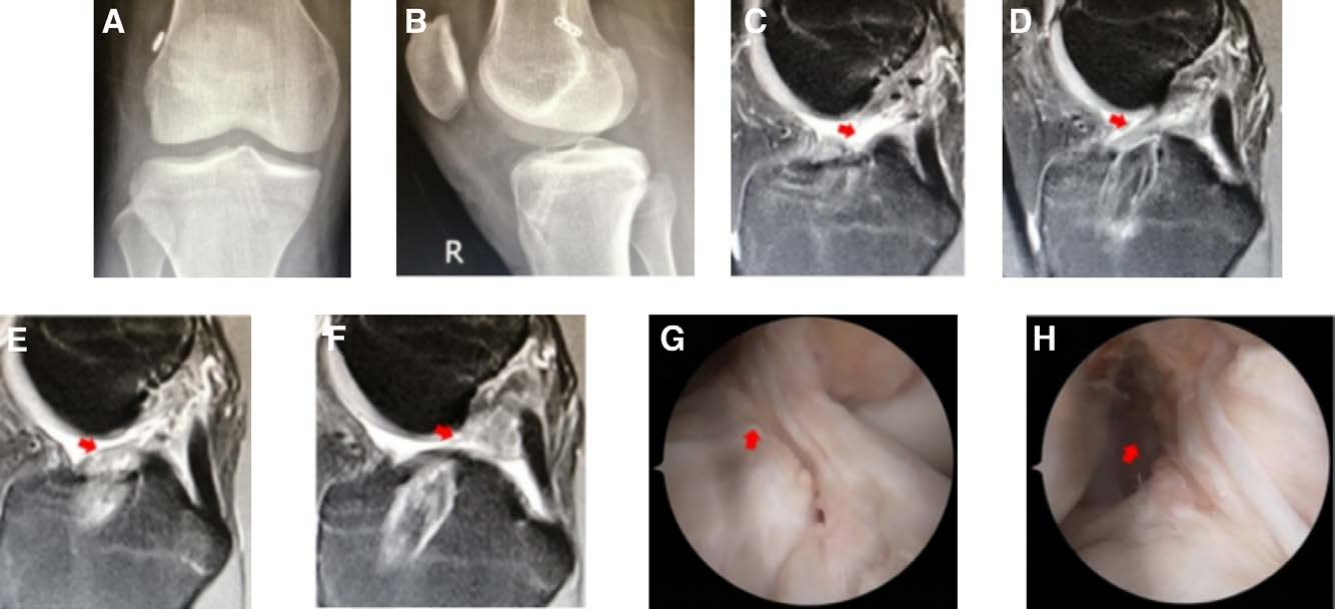
(A and B) DR shows that the tibia and femur tunnel were in good positions. (C and D) MRI findings at half a year: mixed signals of ACL ligament and ACL tendon absorption. (E and F) MR findings at 9 months: mixed signals of ACL ligament and ACL tendon absorption. (G and H) Complete ACL ligament absorption was investigated during revision surgery. ACL = anterior cruciate ligament.

Case 11 involved a 15-year-old male who underwent ACLR in 2020 (allograft, 7 mm) without meniscus management (Fig. 11). One week post-ACLR, symptoms of fever, absence of redness, and swelling appeared in the right knee, with a slightly elevated skin temperature. Despite symptomatic treatment, movement instability persisted, leading to discharge. Subsequent MRIs at 6 months and 9 months showed mixed signals of ACL ligament and tendon absorption, indicating the failure of tendon ligamentization.

#### 3.2.11. Infection

**Figure 12.**
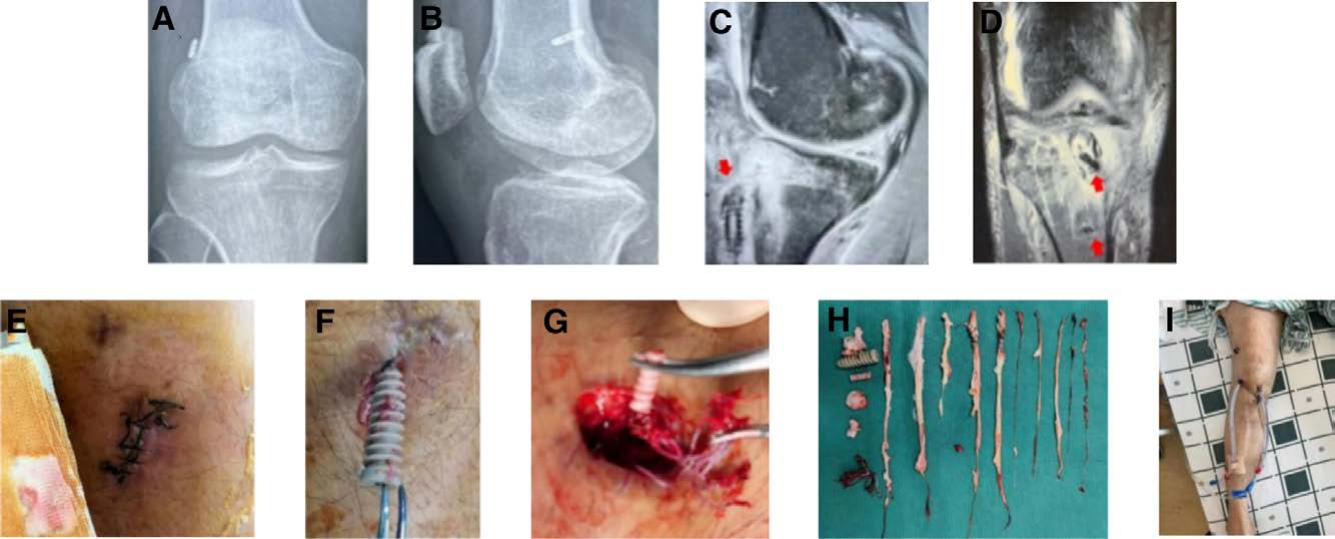
(A and B) DR findings: the femoral and tibial canals were in good positions. (C and D) MRI findings: there was a large amount of inflammation and a notable edema signal in the joint cavity; thus, the tibial bone canal extrusion nail was withdrawn, with an effusion anchor placed below the tibia to fix the suture line of the tendon. (E) The incision where the hamstring tendon of the knee joint was accessed exhibited redness, swelling, and exudation. (F and G) Exudation persisted during the operation at the incision site, necessitating the removal of the tibial extrusion screw and the effusion anchor. (H) The grafted autologous tendon, tibial extrusion screw, and effusion anchor were removed during debridement. (I) The drainage pipe was indwelled for 2 weeks for luminal lavage of the knee joint. When the culture was negative, the lavage was stopped, and the irrigation tube was removed.

Case 12 involved a 36-year-old male who underwent ACLR in another hospital in 2020 (Fig. 12). One week after the operation, the wound at the hamstring tendon site became swollen with exudation. At 6 weeks, their white blood cell count was normal, procalcitonin level was 0.03 ng/mL, C-reactive protein level was 7.69 mg/L, erythrocyte sedimentation rate was 18 mm/h, and bacterial culture of joint fluid was negative. Subsequently, arthroscopy and debridement were performed, the graft and fixation implants were removed, and the bone tunnels were filled with a vancomycin and calcium sulfate mixture. The tissue around the tunnel and wound swab revealed the presence of *Mycobacterium wolinskyi*. Therefore, amikacin was used to irrigate the joint, moxifloxacin was administered intravenously, and clindamycin was administered orally. After successful infection control, revision surgery will be performed in the future.

#### 3.2.12. Multiple ligament injuries leading to failure after ACLR combined with medial collateral ligament (MCL) repair

**Figure 13.**
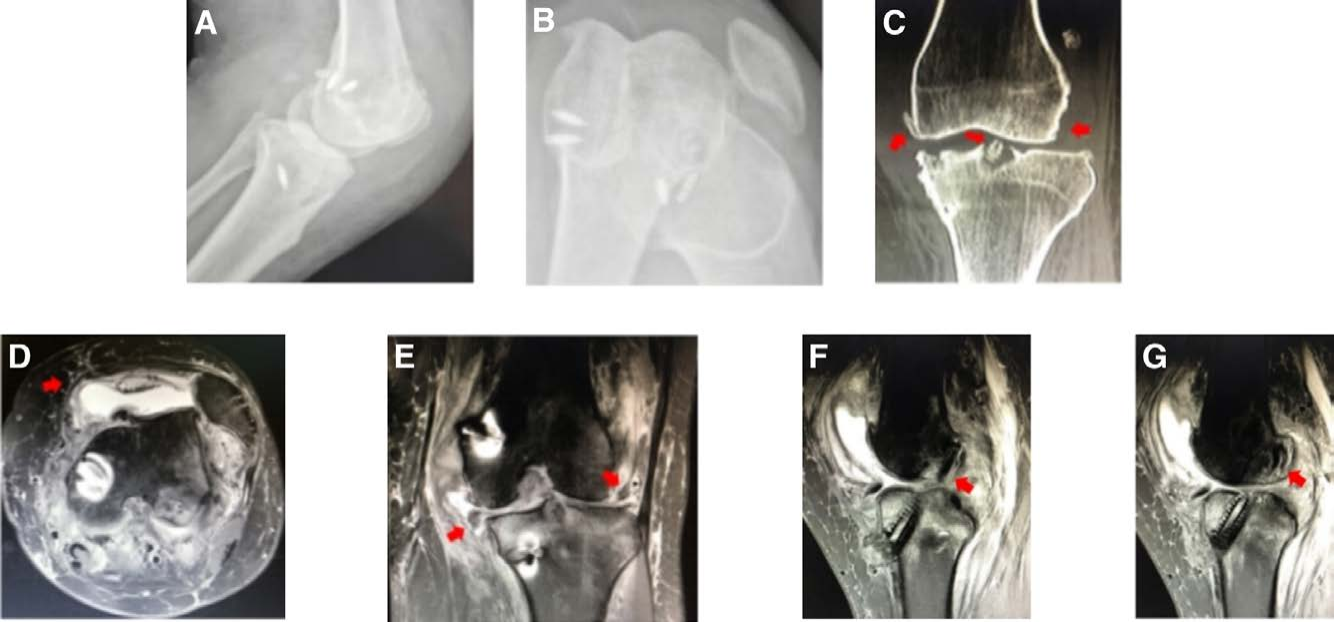
(A and B) DR findings: the left knee joint exhibited subluxation, a posterior tibia drop, and external patella dislocation. (C) CT imaging revealed external patella dislocation of the left knee, obvious degeneration and hyperplasia of the knee joint, the presence of a tibial tunnel extrusion screw protruding into the joint cavity, and subluxation of the knee joint. (D) MRI findings: medial patellofemoral retinacular tear of the left knee. (E) MRI findings revealed subluxation of the left knee joint, a medial collateral ligament tear, medial and lateral meniscus dislocation, and the presence of a tibial tunnel extrusion screw protruding into the joint cavity. (F and G) MRI results: the reconstructed ACL injury of the left knee was edematous; however, some tendons were still visible. ACL = anterior cruciate ligament.

Case 13 involved a 45-year-old female who experienced a car accident resulting in left knee dislocation, ACL, PCL, and MCL rupture, LCL and PT relaxation, lateral meniscus dislocation, a lateral and medial meniscus tear, and dislocation of the patella (Fig. 13). Subsequently, ACLR, meniscus repair, and MCL reconstruction were performed. Two months later, the patient experienced pain and instability in the left knee. MRI revealed subluxation of the left knee joint, medial and lateral dislocation of the meniscus, and the presence of a MCL tear and tibial tunnel extrusion screw that was protruding into the joint cavity. During the revision procedure, the MCL was reconstructed using the semitendinosus tendon, while the LCL and MPFL were sutured and tightened.

## 4. Revision procedure summary

In our revision procedures, we utilized various grafts, including the Ligament Advanced Reinforcement System (LARS) in 10 patients, LARS combined with an allograft in 1 patient, simple allografts in 16 patients, allografts and autografts in 2 patients, and simple autografts in 27 patients. The graft sizes varied among the patients, measuring 6 mm in 5 patients, 7 mm in 21 patients, 8 mm in 24 patients, 9 mm in 5 patients, and 10 mm in 1 patient. Within the group experiencing graft fixation issues, fixation positions were adjusted in 3 patients, and a remnant metal guidewire was removed from 1 patient. Among the patients with infections, debridement was performed in 5 cases. Finally, in the patient with multiple ligament injuries, ACLR, meniscus repair, and MCL injury reconstruction were performed in the initial surgery. Subsequently, during the revision procedure, the MCL was reconstructed using the semitendinosus tendon, while the LCL and MPFL were sutured and tightened.

## 5. Discussion

The number of ACLR surgeries has been rising steadily in recent years. Although numerous long-term studies have indicated that ACLR surgery has a reasonably good success rate and patient satisfaction rate,^[1,3]^ managing the condition as a whole still presents some difficulties for orthopedic surgeons. Overall, the causes of ACLR surgery failure are complex and may be caused by several factors.

The primary cause of ACLR failure often stems from inadequate positioning of the bone tunnel due to surgical technique.^[4]^ To lower the failure rate of ACLR, it remains imperative to position the femoral tunnel correctly. Notably, anatomical reconstruction, rather than early isometric reconstruction, is now the aim of ACLR. Among the various techniques employed, the transtibial tunnel technique is widely favored. While femoral tunnel localization requires more extensive incision and discomfort compared to the placement of both tunnels simultaneously, it is achieved with reduced pain. However, studies emphasize the challenge of precisely determining the position and angle of the femoral stop and anatomical stop, as opposed to the tibial tunnel, which can be more accurately located.^[4–8]^

Another critical component in preventing graft impingement in the intercondylar fossa and PCL is the anatomical positioning of the tibia.^[8]^ Although a North American multicenter consistency study evaluating orthopedic surgeon scores highlighted that the overall consistency in determining the ideal location and size of femoral tunnels (77%) was notably higher compared to the evaluation of tibial tunnels (58%), selecting the optimal location for bone tunnels remains a challenge for orthopedic surgeons.^[5]^

In this study, patients with correctly positioned tunnels commonly reported experiencing severe secondary injuries during revision (54.69%), whereas only 8 patients (12.5%) experienced minor injuries attributed to inadequate recovery following the initial reconstruction. The majority (66.67%) of those with improperly positioned bone tunnels lacked clear precipitating factors and reported trauma-related complaints in 4 cases, contrasting with only a quarter of patients with correctly placed bone canals. Previous research has indicated that patients with improperly positioned tunnels exhibit lower postoperative IKDC and KOOS scores,^[9]^ which may also elucidate our findings: patients with standard bone tunnel placement tend to engage in higher levels of activity and exercise following reconstruction. However, these patients are also more susceptible to trauma, leading to the need for reduced exercise levels among those with improperly positioned tunnels due to poor postoperative recovery.

Currently, autografts, allografts, and synthetic ligaments are the 3 main graft materials used for ACL restoration. Historically, bone-patellar tendon-bone grafts were widely favored and considered the gold standard for ACL reconstruction due to their efficacy.^[10–12]^ However, issues such as anterior knee discomfort and patellar fracture have led to a shift towards hamstring tendon autografts. Notably, although recent 12-year cohort research found a greater risk of rupture following hamstring tendon-based ACL restoration,^[10]^ most studies still find no discernible differences between these types of grafts.^[11–13]^

Allograft tendon usage has seen a rise in recent years, despite research demonstrating that the failure rates for autografts and allografts during primary ACLR range from 6.5% to 7.7% and 6.8% to 8.4%, respectively.^[14]^ Nevertheless, during ACL revision, patients with autografts experienced 2.78 times fewer secondary ruptures within 2 years compared to patients with allografts.^[3]^ Currently, LARS artificial ligaments, with anatomical structures resembling human ligaments, are widely used in ACLR in China. However, due to cost considerations, only some patients still use LARS for primary ACLR. While LARS offers benefits such as anti-fatigue properties and good tissue compatibility, it also presents challenges such as increased bone tunnel pressure and higher ligament and screw hardness, which can cause extrusion nail loosening in patients with osteoporosis who undergo bone tunnel expansion or have bone defects. A histological analysis of failed LARS restorations revealed that the area around the LARS residue displays a typical foreign body reaction, with poor evidence of fibrovascular inward development and peripheral synovial tissue displaying chronic inflammation.^[15]^ Moreover, a mid-to-long-term cohort analysis showed that 8.5% to 33.3% of grafts in patients who underwent LARS repair ruptured or failed.^[16]^ Based on the findings above, careful consideration must be given to selecting LARS as a graft for initial ACL restoration.

In addition to the material or composition of the graft used in ACL restoration, the size of the graft also plays a significant role in ligament–bone healing. Prior to 2017, the grafts typically transplanted in our revision procedures were autografts ranging from 6 to 7 mm in size. However, numerous clinical investigations have shown that grafts with a diameter ranging from 7 to 10 mm will have adequate strength during the middle and late stages of activation, increase knee joint stability, and reduce the chance of revision.^[10,17,18]^ Thus, once we understood this principle, the graft size was increased to 8 or even 9 mm, with the exception of the 7 mm graft used with the LARS.

Injury to the ACL usually alongside damage to the MCL and the meniscus, both of which play crucial roles in knee stability and preventing osteoarthritis.^[18]^ Specifically, after an ACL rupture, the medial meniscus assumes a critical role in regulating tibial advancement. Furthermore, studies have shown that severe medial meniscus abnormalities that go unrepaired may increase the failure rate of reconstruction surgery.^[19–21]^ In our revision procedures, meniscal injuries were identified in 43 cases (77%), indicating the significance of ongoing meniscus damage in patients with failed ACLRs. Previous studies have also indicated that meniscal injury is an independent risk factor for reduced IKDC and KOOS scores after ACLR or revision surgeries.^[20,21]^ Therefore, it is suggested that the meniscus should be explored more carefully to avoid oversight during ACLR procedures.

The most frequent knee joint injury, often accompanying ACL injury, is damage to the MCL.^[22,23]^ Although more than 70% of MCL injuries can heal with excellent nonsurgical treatment, numerous findings still recommend that grade III MCL injuries should be treated concurrently with ACL restoration.^[23,24]^ This simultaneous approach is crucial, as untreated combined MCL injuries can elevate the risk of ACL revision in patients with ACL-MCL injuries. Ultimately, simultaneous repair can considerably improve the medial plane, sagittal plane, and rotation stability of the knee joint.^[25]^ Extraarticular tenodesis is considered a good surgical technique for ACLR and has a good effect on knee joint stability.^[26,27]^ However, none of the patients included in this study whose ACLR failed underwent extraarticular tenodesis as the primary surgical technique, which may be one of the reasons for ACLR revision. Therefore, more research is needed in the future to clarify the clinical value of extraarticular tenodesis for ACLR.

## 6. Limitations

There are some limitations or challenges in this study that need to be accounted for when applying the conclusions of this study. As this study was a retrospective case analysis, we were unable to use a standard method for measuring the tibial slope or the degree of varus or valgus in the limbs before surgery. However, these parameters are crucial for understanding ACLR failures. Therefore, the findings of this study need to be interpreted within the context of the absence of these parameters. Although we categorized and analyzed the causes of ACLR failure, there are limitations in establishing causal relationships between the factors and the failure outcome. As this study is retrospective, it is impossible to completely rule out the influence of other potential confounding factors. Thus, we cannot definitively prove that a specific factor is the direct cause of ACLR failure but can only indicate associations between them. Therefore, future research with larger sample sizes and clinical data that exclude potential confounders is needed to validate the possible causes of failure proposed in this study.

## 7. Conclusion

The success of ACL restoration hinges on the meticulous management of various factors, including the treatment of combined injuries, surgical technologies employed, and graft and fixation methods selected. Notably, particular attention should be paid to the surgical technology of the bone tunnel site among these. Overall, the success rate and therapeutic effectiveness of arthroscopic ACLR depend on the effective management of these relevant elements.

## Author contributions

**Conceptualization:** Xiao Jin, Jianke Pan.

**Data curation:** Xiao Jin, Meiping Yang, Jinlong Zhao, Jianke Pan.

**Formal analysis:** Meiping Yang, Jinlong Zhao.

**Investigation:** Xiao Jin, Zihan Lin, Jinlong Zhao, Guihong Liang.

**Methodology:** Xiao Jin, Jianke Pan.

**Project administration:** Jianke Pan.

**Resources:** Weiyi Yang.

**Software:** Lingfeng Zeng.

**Supervision:** Weiyi Yang.

**Validation:** Lingfeng Zeng, Jun Liu.

**Writing – original draft:** Xiao Jin, Zihan Lin, Meiping Yang, Jinlong Zhao, Lingfeng Zeng, Jun Liu, Guihong Liang, Weiyi Yang, Jianke Pan.

**Writing – review & editing:** Xiao Jin, Zihan Lin, Meiping Yang, Jinlong Zhao, Lingfeng Zeng, Jun Liu, Guihong Liang, Weiyi Yang, Jianke Pan.
